# Reduced incidence of Crohn’s disease in systemic sclerosis: a nationwide population study

**DOI:** 10.1186/s12891-015-0693-0

**Published:** 2015-09-14

**Authors:** Chia-Chun Tseng, Jeng-Hsien Yen, Wen-Chan Tsai, Tsan-Teng Ou, Cheng-Chin Wu, Wan-Yu Sung, Ming-Chia Hsieh, Shun-Jen Chang

**Affiliations:** Graduate Institute of Clinical Medicine, College of Medicine, Kaohsiung Medical University, Kaohsiung, Taiwan; Division of Rheumatology, Department of Internal Medicine, Kaohsiung Medical University Hospital, Kaohsiung, Taiwan; Institute of Biomedical Sciences, National Sun Yat-sen University, Kaohsiung, Taiwan; Graduate Institute of Medicine, College of Medicine, Kaohsiung Medical University, Kaohsiung, Taiwan; Division of Endocrinology and Metabolism, Department of Internal Medicine, Changhua Christian Hospital, Changhua, Taiwan; Graduate Institute of Integrated Medicine, China Medical University, Taichung, Taiwan; Department of Kinesiology, Health and Leisure Studies, National University of Kaohsiung, Kaohsiung, Taiwan

## Abstract

**Background:**

To date, there has been no studies to evaluate the incidence of Crohn’s disease in systemic sclerosis patients. The goals of this study were to evaluate the incidence of Crohn’s disease and its relationship with sex and age in patients with systemic sclerosis.

**Methods:**

We enrolled patients with systemic sclerosis and controls from Taiwan’s Registry of Catastrophic Illness Database and National Health Insurance Research Database. Every systemic sclerosis patient was matched to at most three controls by sex, age, month and year of initial diagnosis of systemic sclerosis. The standardized incidence ratio (SIR) of Crohn’s disease in systemic sclerosis patients, and 95 % confidence interval (95 % CI) were calculated. Cox hazard regression was used to calculate the hazard ratio (HR).

**Results:**

The study enrolled 2,829 patients with systemic sclerosis and 8,257 controls. Male and female patients with systemic sclerosis both had lower rates of incident Crohn’s disease (SIR: 0.18, 95 % CI = 0.05–0.62; SIR: 0.10, 95 % CI = 0.05–0.21, respectively). The risk of incident Crohn’s disease in systemic sclerosis was still lower than in controls when we stratified the patients according to their ages. In Cox hazard regression, the hazard rates of Crohn’s disease were lower in systemic sclerosis patients after adjusting for genders and ages (HR: 0.12, 95 % CI = 0.06–0.21, *p* < 0.001).

**Conclusions:**

Systemic sclerosis is associated with decreased incidence of, irrespective of sex and age of the patients.

## Background

Systemic sclerosis is a multisystem autoimmune disease characterized by endothelial dysfunction, widespread obliterative vasculopathy, and excessive collagen deposition in affected organs, culminating in tissue fibrosis [1]. Immunologically, it is associated with autoantibodies [1]. Clinically, it is divided into a limited form and a diffuse form [1]. Besides its own morbidity, there is an increasingly number of reports showing coexisting medical diseases such as systemic lupus erythematosus [2] and autoimmune thyroid disease associated with systemic sclerosis [3]. However, there are also studies pointing to a reduced risk of autoimmune diseases, such as inflammatory bowel disease, in association with systemic sclerosis [4].

Crohn’s disease is a type of inflammatory bowel disease. Several studies have varied conclusions about whether systemic sclerosis implies a greater risk of Crohn’s disease. For example, in one study [5], the risk of Crohn’s disease differed across two databases in systemic sclerosis patients. Another study showed reduced risk of inflammatory bowel disease in the family members of patients with systemic sclerosis [4]. Besides conflicting results in the literature, previous studies focused on the prevalence of Crohn’s disease instead of the incidence of Crohn’s disease. In addition, the relationship of age and sex in regards to Crohn’s disease incidence in systemic sclerosis needs to be further elucidated. Thus we conducted this study to explore the incidence of Crohn’s disease and its relationship with sex and age in systemic sclerosis patients.

## Methods

### Data resources

In Taiwan, the Ministry of Health and Welfare implemented the National Health Insurance (NHI) program in 1995. Since then, 99.6 % of the total population in Taiwan has enrolled in the NHI [6]. The Ministry also established the NHI database which has collected the dates of birth, the diagnoses of patients, the dates of doctor’s visits, sex, and other related information of patients. Numerous studies utilize this database to survey the risk of comorbidities in diseases including systemic sclerosis and Crohn’s disease [7, 8].

The NHI medical claims database is administered by Taiwan’s National Health Research Institutes (NHRI). The NHRI provided National Health Insurance Research Database (NHIRD) 1,000,000 random subjects for this study, which represents approximately five percent of Taiwan’s population. A systematic, random sampling method was used to build this representative database. There were 23 million patients in the original claim database, with one specific corresponding identification number for each person. A random number generator generated 1,000,000 random numbers. After eliminating duplicate values, we used these random numbers to include persons with corresponding identification numbers in NHIRD. The process was repeated until 1,000,000 subjects were enrolled in NHIRD. This population is representative of Taiwan’s national population with respect to age, sex, and other characteristics. For this study, we obtained a longitudinal cohort from the NHIRD from 1998 to 2011. Another database acquired from the NHRI was the Registry of Catastrophic Illness that included information of all 23 million Taiwanese citizens. According to Taiwan’s regulations, insured persons with major diseases such as systemic sclerosis and Crohn’s disease could apply for a catastrophic illness certificate for exemption from copayment. For Crohn’s disease to be registered in the Registry of Catastrophic Illness database, the attending physician must submit information about the clinical diagnosis, laboratory results, endoscopic findings, pathological reports as well as the possible image studies to register the patient’s diagnosis of Crohn’s disease, as well as for excluding intestinal tuberculosis. For systemic sclerosis to be registered in the Registry of Catastrophic Illness database, patients must fullfill 1980 Ammerican College of Rheumatology criteria. These applications for catastrophic illness certificates were reviewed by related specialists and justified by a meticulous examination of the medical records as well as related exam results. Because the NHRI releases these two databases with scrambled identification numbers, it is impossible to identify patients from these databases. No written informed consent was necessary in this situation according to Taiwan’s regulations. Thus the analysis of the databases was exempt from the ethical approval by the Institutional Review Board of Kaohsiung Medical University Hospital (KMUH-IRB-EXEMPT-20140007).

### Study sample

The NHI database diagnosis coding mirrors the International Classification of Diseases, Ninth Revision (ICD-9), Clinical Modification diagnosticcriteria. We used Registry of Catastrophic Illness to identify patients with systemic sclerosis (ICD-9: 7101) diagnosed in 1998 to 2011 because patients diagnosed with systemic sclerosis before this period might already have systemic sclerosis with unknown duration. We excluded patients with Crohn’s disease (ICD-9: 555) diagnosed before systemic sclerosis or within 1 year after diagnosis of systemic sclerosis. The reason behind excluding patients with Crohn’s disease diagnosed within 1 year after diagnosis of systemic sclerosis is to ensure that Crohn’s disease diagnosis came after their diagnosis of systemic sclerosis. Only patients older than 20 years old were included in our analysis. We used NHIRD to collect the control group consisting of patients without systemic sclerosis. Control patients could have other diseases except systemic sclerosis, such as glaucoma, hyperlipidemia, and so on. Subjects with preexisting Crohn’s disease prior to enrollment or within one year after enrollment were excluded. Each systemic sclerosis patient was matched to at most three control patients by gender, year of birth (within 2 years of birthdates of cases), and the time of first diagnosis at enrollment. The goal of this matching was to ensure that there were no significant difference in sex, age, and follow-up time between study subjects and controls.

### The endpoint and follow up

Follow-up started on the date of first diagnosis of systemic sclerosis in the systemic sclerosis group and on the same date in the matched control group and ended on the date of diagnosis of Crohn’s disease (identified with ICD-9: 555 in the Registry of Catastrophic Illness database), death, transfer out, or the end of 2011.

### The Incidence of Crohn’s disease and Standardized Incidence Ratio (SIR)

We estimated the incidence of Crohn’s disease per 1000 person-years in patients with systemic sclerosis and control subjects, and calculated the age-adjusted standardized incidence ratio (SIR), which is the ratio between observed number of Crohn’s disease and expected number of Crohn’s disease, calculated as observed cases divided by expected cases for each age group. The well-described method of 95 % confidence intervals (95 % CI) estimation [9] was used. We also defined the onset age of Crohns’ disease as the age at which the patient received the diagnosis of Crohn’s disease in the Registry of Catastrophic Illness database.

### Statistical analysis

Age-adjusted SIR of Crohn’s disease in different groups of systemic sclerosis was calculated. We used Student’s *t*-test to compare the onset age of Crohn’s disease between different groups of patients. We used the Kaplan-Meier analysis to estimate the event-free probability of Crohn’s disease after enrollment. The log-rank test was used to compare among different groups of patients. We utilized Cox hazard regression to calculate the hazard ratio (HR) and 95 % confidence intervals (95 % CI) for incident Crohn’s disease in patients with systemic sclerosis compared to controls. All statistical operations were performed using SPSS (v19.3) after mining the national outpatient records using the PERL (v5.8).

## Results

For the analysis of risk of Crohn’s disease, 2,829 patients with systemic sclerosis and 8,257 controls were included. All patients were gathered from the outpatient database from 1998 to 2011. The mean ages of case and control groups were 49.27 ± 14.63 and 49.44 ± 14.57 years, respectively (Table 1). The age distribution was not statistically different between systemic sclerosis patients and controls (*p* > 0.05).

**Table 1:** The age distribution in baseline and follow-up time of systemic sclerosis patients and the controls

	Systemic sclerosis		Controls	
	(*n =* 2829)		(*n =* 8257)	
Age(years; mean ± SD)	49.27 ± 14.63		49.44 ± 14.57	
Age group at enrollment(years)	*n* (%)	Person-years^a^	*n* (%)	Person-years
> = 20, <30	315 (11.13)	2105.67	897 (10.86)	6676.17
> = 30, <40	459 (16.22)	3596.42	1301 (15.76)	11209.67
> = 40, <50	697 (24.64)	5448.33	2067 (25.03)	18276.25
> = 50, <60	681 (24.07)	4562.83	1975 (23.92)	15573.33
> = 60, <70	427 (15.09)	2607.00	1294 (15.67)	9607.75
> = 70, <80	220 (7.78)	1161.83	636 (7.70)	4753.08
> = 80	30 (1.06)	118.67	87 (1.05)	532.50

*SD* standard deviation; ^a^:The total follow-up person-years

### Reduced risk of Crohn’s disease in systemic sclerosis

A total of 332 cases of incidental Crohn’s disease were diagnosed during the follow-up period, 11 in the case group and 321 in the control group (Table 2). The incidence of Crohn’s disease was estimated to be 0.56 cases per 1000 person-years among systemic sclerosis cases during the 1998–2011 period; controls had an incidence of 4.82 cases per 1000 person-years. Age-adjusted SIR of Crohn’s disease was 0.12 (95 % CI = 0.06–0.22), which suggests that patients with systemic sclerosis had 0.12 times of the risk of developing Crohn’s disease compared to controls after adjusting for age. Furthermore, the risk of incident Crohn’s disease in systemic sclerosis patients was lower than controls when stratified based on their respective ages.

**Table 2:** Age-adjusted standardized incidence ratio of Crohn’s disease in scleroderma patients stratified by age

	Systemic sclerosis	Control	Incidence Ratio	95 % CI
	(*n =* 2829)	(*n =* 8257)		
Age at diagnosis	Observed(Inc)^a^	Observed(Inc)		
> = 20, <30	1 (0.47)	39 (5.84)	0.08	0.01–0.58
> = 30, <40	4 (1.11)	51 (4.55)	0.24	0.09–0.66
> = 40, <50	2 (0.37)	77 (4.21)	0.09	0.02–0.37
> = 50, <60	3 (0.66)	72 (4.62)	0.14	0.04–0.44
> = 60	1 (0.26)	82 (5.51)	0.05	0.01–0.36
Total	11 (0.56)	321 (4.82)	0.12	0.07–0.22
Age adjusted standardized incidence ratio			0.12	0.06–0.22

*95 % CI* 95 % confidence interval; ^a^:observed numbers of new diagnosed Crohn’s disease (incidence per 1000 person-years)

We then stratified patients according to their sex. There were a total of 514 male patients with systemic sclerosis and 1,448 male controls (Table 3), 2,315 female patients with systemic sclerosis and 6,809 female controls (Table 4).

**Table 3:** Age-adjusted standardized incidence ratio of Crohn’s disease in male scleroderma patients stratified by age

	Systemic sclerosis	Control	Incidence Ratio	95 % CI
	(*n =* 514)	(*n =* 1448)		
Age at diagnosis	Observed(Inc)^a^	Observed(Inc)		
> = 20, <40	1 (1.31)	16 (7.03)	0.19	0.03–1.43
> = 40, <50	1 (1.59)	9 (4.21)	0.38	0.05–2.97
> = 50	1 (0.59)	37 (5.35)	0.11	0.02–0.79
Total	3 (0.97)	62 (5.47)	0.18	0.06–0.57
Age adjusted standardized incidence ratio			0.18	0.05–0.62

*95 % CI* 95 % confidence interval; ^a^:observed numbers of new diagnosed Crohn’s disease (incidence per 1000 person-years)

**Table 4:** Age-adjusted standardized incidence ratio of Crohn’s disease in female scleroderma patients stratified by age

	Systemic sclerosis	Control	Incidence Ratio	95% CI
	(*n=*2315)	(*n=*6809)		
Age at diagnosis	Observed(Inc)^a^	Observed(Inc)		
>=20, <30	1 (0.56)	35 (6.07)	0.09	0.01–0.65
>=30, <40	3 (0.95)	39 (3.96)	0.24	0.07–0.78
>=40, <50	1 (0.21)	68 (4.21)	0.05	0.01–0.36
>=50, <60	2 (0.53)	57 (4.60)	0.12	0.03–0.49
>=60	1 (0.33)	60 (5.38)	0.06	0.01–0.44
Total	8 (0.48)	259 (4.68)	0.10	0.05–0.20
Age adjusted standardized incidence ratio			0.10	0.05–0.21

*95 % CI* 95 % confidence interval; ^a^:observed numbers of new diagnosed Crohn’s disease (incidence per 1000 person-years)

A total of 65 cases of incidental Crohn’s disease in male patients were diagnosed during the follow-up period, 3 in the case group and 62 in the control group. The incidence of Crohn’s disease was estimated to be 0.97 cases per 1000 person-years among male systemic sclerosis cases during the 1998–2011 period; male controls had an incidence of 5.47 cases per 1000 person-years (Table 3). Age-adjusted SIR of Crohn’s disease was 0.18 (95 % CI = 0.05–0.62), which suggests male patients with systemic sclerosis had 0.18 times of the risk of developing Crohn’s disease compared to male controls after adjusting for age. Reduced risk of Crohn’s disease only existed in patients older than 50 years due to a small sample size in male patients younger than 50 years.

A total of 267 cases of incidental Crohn’s disease in female patients were diagnosed during the follow-up period, 8 in the case group and 259 in the control group. The incidence of Crohn’s disease was estimated to be 0.48 cases per 1000 person-years among female systemic sclerosis cases during the 1998–2011 period; female controls had an incidence of 4.68 cases per 1000 person-years (Table 4). Age-adjusted SIR of Crohn’s disease was 0.10 (95 % CI = 0.05–0.21), which suggests that female patients with systemic sclerosis had 0.10 times of the risk of developing Crohn’s disease compared to female controls after adjusting for age. Furthermore, reduced risk of Crohn’s disease in systemic sclerosis was present when stratified based on their respective ages.

### The onset age of Crohn’s disease

We analyzed the onset age of Crohn’s disease according to sex. In male patients, the mean onset age of Crohn’s disease in systemic sclerosis patients and control groups were 50.22 ± 10.35 years and 57.11 ± 15.04 years (data not shown), and not significantly different (*p =* 0.438). In female patients, the mean onset age of Crohn’s disease in systemic sclerosis patients and control groups were 46.89 ± 13.57 years and 53.10 ± 14.38 years (data not shown), and not significantly different (*p =* 0.229).

### The hazard ratio of Crohn’s disease in systemic sclerosis patients

We then used Cox hazard regression to calculate the hazard ratio of Crohn’s disease among all participants after adjusting for age, sex, and systemic sclerosis. Systemic sclerosis (HR = 0.12, 95 % CI = 0.06–0.21, *p* < 0.001) was significantly associated with reduced incident Crohn’s disease after adjusting for age and sex.

**Fig. 1:**
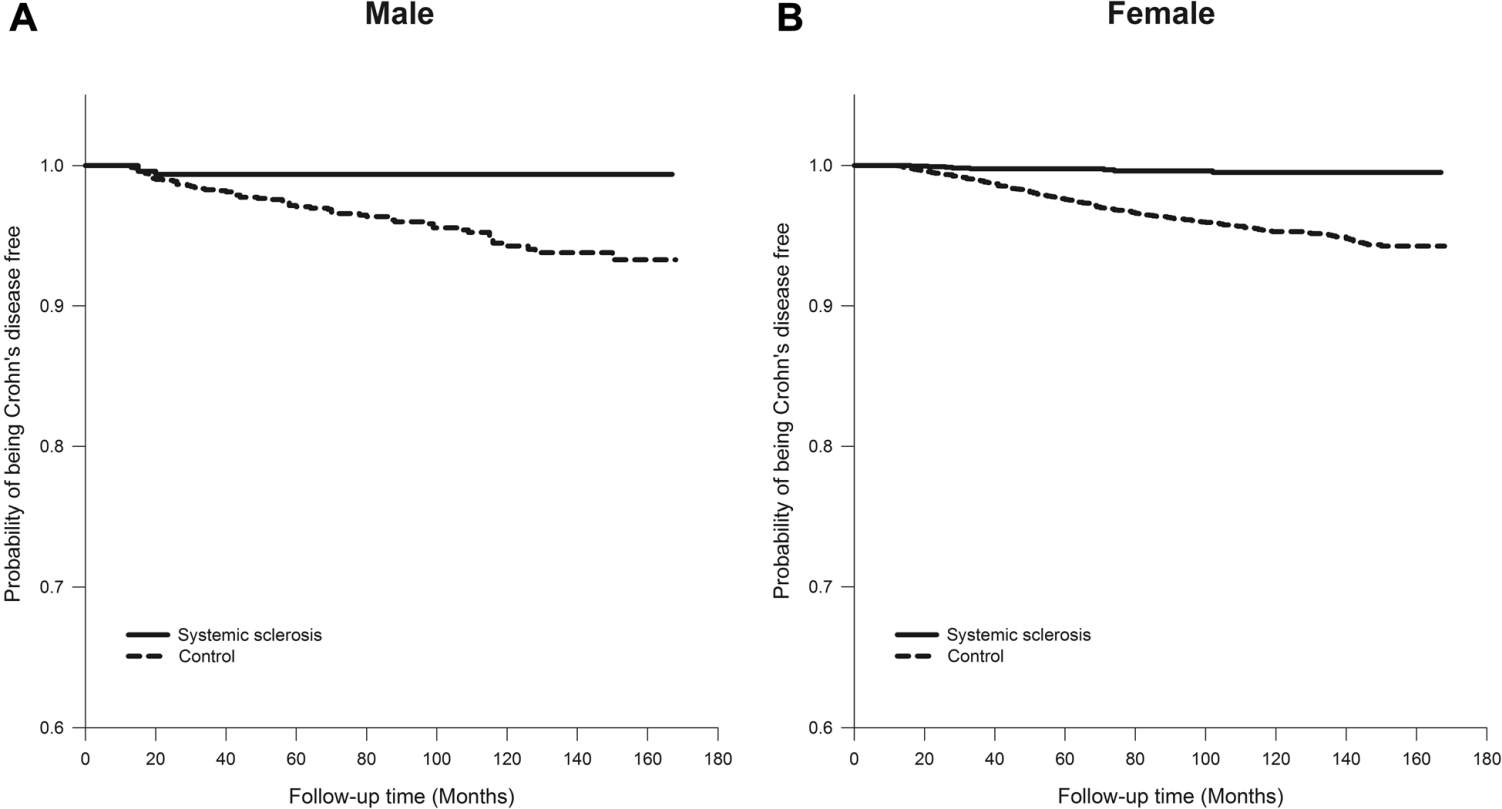
Kaplan-Meier plot of Crohn’s disease for patients with systemic sclerosis and controls. **a** For male systemic sclerosis patients and male controls (*p* < 0.001, estimated by the log-rank test). **b** For female systemic sclerosis patients and female controls (*p* < 0.001, estimated by the log-rank test)

A comparison of the risk of Crohn’s disease between systemic sclerosis patients and control patients is shown in Fig. 1. The probability of developing Crohn’s disease were lower in male systemic sclerosis patients than in male controls (*p* < 0.001). Female systemic sclerosis patients also had lower probability of developing Crohn’s disease compared to female controls (*p* < 0.001).

## Discussion

In this study, we utilized a retrospective cohort study to demonstrate significantly reduced incidence of Crohn’s disease in patients with systemic sclerosis, irrespective of gender and age of the patient. The age of diagnosis of Crohn’s disease was not different between systemic sclerosis patients and controls. To our knowledge, this was the first study to demonstrate reduced incidence of Crohn’s disease in systemic sclerosis. Past studies were discrepant in the relationship between systemic sclerosis and Crohn’s disease. For example, one study in French [4] noted decreased prevalence of inflammatory bowel disease in family members of systemic sclerosis patients. However, the diagnosis of Crohn’s disease was identified by questionnaire rather than validated databases, as in our study. Another study [5] found conflicting results about risk of Crohn’s disease in systemic sclerosis across two different healthcare databases. Aside from unrepresentational sample, rather short follow-up time (2 years) was present. Furthermore, no information about incidence was available from these two studies.

The reason behind this phenomenon was not immediately clear from this epidemiology study. There were no studies to explore the protective effects of systemic sclerosis on Crohn’s disease. However, genome-wide association studies and immunologic studies implicated genetic and immunologic factors in the two diseases.

The frequency of the *HLA-DR3* serotype was higher in patients of systemic sclerosis [10, 11] but lower in Crohn’s disease [12]. On the other hand, the presence of the *HLA-DR7* serotype was correlated with decreased risk of systemic sclerosis [13], but was associated with higher risk of Crohn’s disease [12]. It was hypothesized that the specific *HLA* may determine the targeting of particular antigens [14], resulting in specific associations with different diseases.

Besides *HLA*, *protein tyrosine phosphatase non-receptor type 22* (*PTPN22*) was implicated in both systemic sclerosis and Crohn’s disease. Previous study showed *PTPN22* C1858T (R620W), a gain of function [15], was correlated with higher risk of systemic sclerosis [16–20], but reduced risk of Crohn’s disease [18, 21].

Past studies showed reduced PTPN22 expression in the gastrointestinal tract of patients with Crohn’s disease [22], and PTPN22 deficiency caused: increased secretion of interleukin-6 (IL-6) and IL-8, increased activation of p38 and c-Jun N-terminal kinase (JNK), increased phosphorylation of nuclear factor κB (NF-kB) p65, and increased autophagy, all which could lead to prolonged survival of activated macrophage and promote inflammatory condition in Crohn’s disease [23]. Thus *PTPN22* C1858T polymorphism, which is associated with systemic sclerosis susceptibility, reduced the risk of Crohn’s disease.

In addition to *HLA* and *PTPN22*, immunologic factors were also involved. T-bet, encoded by the *TBX21* gene, was involved in systemic sclerosis and Crohn’s disease. In the animal model of systemic sclerosis, T-bet knockout mice were associated with increased susceptibility to skin sclerosis [24]. *TBX21* rs11650354 was also a susceptibility factor in systemic sclerosis [25]. On the contrary, enhanced T-bet expression in active Crohn’s disease intestine was demonstrated [26]. Higher T-bet expression in peripheral blood mononuclear cells in Crohn’s disease was also noted [27]. T-bet deficiency protects mice from colitis, but overexpression of T-bet accelerates colitis [28]. T-bet up-regulation established immunopathology in Crohn’s disease [29]. Thus T-bet deficiency in systemic sclerosis may be a cause in reducing the risk of Crohn’s disease.

There were some limitations in this study. First, antibiotics, nonsteroidal antiinflammatory drugs (NSAIDs), smoking, and appendectomy were not adjusted in this study. However, prednisolone, azathioprine, cyclophosphamide, mycophenolate mofetil, methotrexate, and penicillamine, which were used to treat patients with systemic sclerosis, were associated with infection requiring antibiotics treatment [30–33]. Antibiotics increased rather than decreased incidence of Crohn’s disease [34]. NSAIDs, which were used in in systemic sclerosis, increased instead of decreasing subsequent Crohn’s disease [35]. In addition, although smoking and appendectomy increased risk of Crohn’s disease in western contries [36, 37], smoking and appendectomy were not risk factors for Crohn’s disease in Asia [36, 38]. Thus antibiotics, nonsteroidal antiinflammatory drugs (NSAIDs), smoking, and appendectomy couldn’t explain the protective effects exerted by systemic sclerosis on Crohn’s disease. Second, systemic sclerosis was divided into a limited form and a diffuse form. However, both shared the same ICD-9 code. It was noted in previous studies that different disease subtypes implied different clinical courses [39]. Patients couldn’t be stratified further based on systemic sclerosis subtypes—limited form and diffuse form—because patients with different subtypes had similar ICD-9 diagnosis code in the Registry of Catastrophic Illness database. Furthermore, information about family history and diet style was not available from the national health insurance database. These common limitations have been noted in the claim-based dataset [8].

## Conclusion

In conclusion, this was the first nationwide population-based study to demonstrate reduced incidence of Crohn’s disease in patients of systemic sclerosis. Current literature shed some light on some potential causes behind this phenomenon, including *HLA*, *PTPN22*, and T-bet. However, further studies are necessary to clarify the risk of Crohn’s disease in systemic sclerosis in regards to different subtypes of scleroderma.

## Abbreviations

SIR: Standardized incidence ratio
95 % CI: 95 % confidence interval
HR: Hazard ratio
NHI: National Health Insurance
NHRI: National Health Research Institutes
NHIRD: National Health Insurance Research Database
ICD-9: International Classification of Diseases, Ninth Revision
PTPN22: protein tyrosine phosphatase non-receptor type 22
IL: Interleukin
JNK: c-Jun N-terminal kinase
NF-κB: Nuclear factor κB
NSAIDs: Nonsteroidal antiinflammatory drugs
